# Validation and prognostic utility of SLERPI in a single-centre retrospective Central European cohort

**DOI:** 10.1177/09612033261474985

**Published:** 2026-08-05

**Authors:** Andrea Zoli, Seda Nur Aydogdu, Danae-Mona Nöthling, Paraskevi Chasani, Maria Antonietta D' Agostino, Silvia Laura Bosello, Andreas Ramming, Christina Bergmann, Georg Schett, Chiu Wai Shirley Chan, Panagiotis Garantziotis

**Affiliations:** 1 Department of Internal Medicine 3-Rheumatology and Immunology, Friedrich-Alexander-Universität (FAU) Erlangen-Nürnberg and Universitätsklinikum Erlangen, Erlangen, Germany; 2 Deutsches Zentrum Immuntherapie (DZI), FAU Erlangen-Nürnberg and Universitätsklinikum Erlangen, Erlangen, Germany; 3 Division of Rheumatology, IRCCS-Università Cattolica del Sacro Cuore, 18654 Fondazione Policlinico Universitario A. Gemelli, Rome, Italy; 4 Department of Neuropathology, Friedrich-Alexander-Universität (FAU) Erlangen-Nürnberg and Universitätsklinikum Erlangen, Erlangen, Germany; 5 Division of Rheumatology and Clinical Immunology, Department of Medicine, 25809 University of Hong Kong, Hong Kong, Hong Kong

**Keywords:** systemic lupus erythematosus, SLERPI, classification criteria, validation, disease flare

## Abstract

**Objectives:**

To evaluate the diagnostic performance of the SLE Risk Probability Index (SLERPI) in a European cohort and explore its potential prognostic utility for early flare risk stratification.

**Methods:**

This retrospective study included 280 patients with physician-diagnosed SLE and 156 non-SLE controls from a tertiary centre. Diagnostic performance (sensitivity, specificity, and area under the curve [AUC]) of the SLERPI and the 2019 European League Against Rheumatism (EULAR)/American College of Rheumatology (ACR) classification criteria were assessed against physician diagnosis as the reference standard. Severe flares within 3 years of diagnosis were defined using the SELENA-SLEDAI Flare Index. Multivariable logistic regression was used to identify predictors of severe flare.

**Results:**

SLERPI demonstrated a higher AUC than the 2019 EULAR/ACR criteria (0.967, 95% CI 0.950–0.984 vs 0.932, 95% CI 0.908–0.957; DeLong p = 0.0004) and greater specificity (97.4% vs 94.9%), whereas sensitivity (85.7% vs 87.1%) and negative predictive value (79.2% vs 80.4%) were slightly lower. Analysis of discordant classifications revealed that the SLERPI captured cutaneous-predominant presentations more effectively, while the EULAR/ACR criteria showed greater sensitivity for serositis-driven disease. Patients who developed a severe flare within 3 years had significantly higher baseline SLERPI scores (*p* = 0.047). Youden’s J statistic identified a threshold of ≥10.2 points as potentially informative for severe flare risk stratification. In multivariable analysis, a model incorporating SLERPI constituent items identified proteinuria and serositis at diagnosis as independent predictors of severe flare (AUC 0.708, 95% CI 0.641-0.775); bootstrap internal validation yielded a lower, optimism-corrected AUC of 0.635 (95% CI 0.574-0.695).

**Conclusion:**

SLERPI demonstrates high diagnostic accuracy in a single-centre retrospective Central European cohort. Exploratory analyses suggest that higher baseline SLERPI scores may be associated with an increased risk of early severe flares; however, these findings require external validation before prognostic use can be recommended.

## Significance and innovations

• SLERPI demonstrated excellent diagnostic accuracy in a single-centre retrospective Central European cohort.
• Preliminary analysis suggested a weak association between SLERPI and risk of severe flare within 3 years of diagnosis, warranting further investigation.

## Introduction

Systemic lupus erythematosus (SLE) is a multisystem autoimmune disease characterized by clinical heterogeneity and a complex immunological landscape.^1^ The diagnosis of SLE remains a clinical judgment based on the recognition of typical clinical and immunological features, as well as the exclusion of mimicking entities such as other rheumatological or infectious diseases.^2^ In the absence of definitive diagnostic criteria, clinicians often rely on diagnostic evaluations referencing established classification criteria, which are developed to standardize patient recruitment in clinical research.^3,4^ Based on machine learning, the SLE Risk Probability Index (SLERPI) has recently been developed as a diagnostic model to produce individualized SLE risk probabilities, similar to clinical reasoning.^5^ Using a predefined cut-off score for dichotomous classification, the SLERPI demonstrated high accuracy for SLE in multiple patient cohorts.^5,6,7^ Validation in diverse populations and assessment of its longitudinal application will further enhance the robustness of the model for clinical use.

Most patients with SLE have a relapsing-remitting disease course, yet individual risk of disease flare remains difficult to predict. Disease flares independently increase the risk of irreversible organ damage, highlighting the importance of risk stratification strategies to identify patients at risk of disease flare early on, ideally at the time of diagnosis. The European League Against Rheumatism (EULAR)/American College of Rheumatology (ACR) 2019 classification criteria use a domain-based assessment with variable weights assigned to different clinical items.^8^ Patients with a high EULAR/ACR criteria score have been associated with a more active disease course over the first 5 years.^9^ The potential of SLERPI, a simple scoring system based on weighted clinical items, for prognostic stratification in addition to its diagnostic utility remains to be explored.

In this study, we evaluated the classification performance of SLERPI in a a single-centre retrospective Central European SLE cohort. We also explored the utility of SLERPI for risk stratification of severe disease flare within 3 years of diagnosis.

## Material and methods

### Study design and patient population

This retrospective study included patients with physician-diagnosed SLE between 1990 and 2000 registered at Universitätsklinikum Erlangen, along with non-SLE controls diagnosed with other rheumatic diseases during the same period.

Baseline clinical and serological parameters were extracted from medical records at the time of the first rheumatological evaluation. Data were collected to determine fulfilment of the 2019 European League Against Rheumatism/American College of Rheumatology (EULAR/ACR) classification criteria^8^ and to calculate the SLERPI across its 14 domains.^5^ The EULAR/ACR classification criteria were applied using a threshold score of ≥10 points as per the original publication.^8^ The SLERPI was calculated as previously described, with a score of >7 used to classify patients as having SLE.^5^

Disease flares occurring within 3 years of diagnosis were recorded retrospectively by a rheumatologist using the Safety of Estrogen in Lupus Erythematosus - SLE Disease Activity Index Flare Index (SELENA-SLEDAI Flare Index)^10^ and classified as severe or non-severe according to its predefined criteria.^10^ As flare ascertainment and SLERPI scoring were performed by the same assessor(s), formal blinding to SLERPI status was not implemented for this retrospective analysis.

Patients with missing flare follow-up data were excluded from the prognostic analyses.

### Statistical analysis

Continuous variables are presented as mean and standard deviation (SD) and categorical variables as number and percentage. Between-group comparisons for continuous variables were performed using the Mann-Whitney U test and for categorical variables using the chi-square test. Age was missing in 5 of 280 SLE patients (1.8%) and was imputed using the mean age of the SLE group; no other variable used in the diagnostic analyses had missing data.

The diagnostic performance of the SLERPI and the EULAR/ACR classification criteria was evaluated by calculating sensitivity, specificity, positive predictive value (PPV), and negative predictive value (NPV). Receiver operating characteristic (ROC) curves were constructed for both instruments using their continuous scores and the areas under the curve (AUC) were compared using the DeLong method for paired ROC curves.

For the prognostic analysis, SLERPI items with non-zero variance were subsequently entered into a multivariable logistic regression model to identify independent predictors of severe flare, with results expressed as adjusted odds ratios (OR) with 95% confidence intervals (CI). The discriminatory capacity of the multivariable model was assessed by AUC. The optimal prognostic threshold of the continuous SLERPI score for predicting severe flare was identified using Youden’s J statistic (sensitivity + specificity −1).

Internal validation of the multivariable model was performed using bootstrap resampling (1000 replicates), with optimism-corrected estimates of discrimination (AUC) and calibration (slope and intercept) calculated using Harrell’s method.

All analyses were performed in R using the following packages: dplyr, pROC, ggplot2, patchwork, gtsummary, rms, and broom. A two-sided p-value of <0.05 was considered statistically significant throughout.

## Results

### Study cohort and patient characteristics

A total of 436 patients were included (280 SLE, 156 non-SLE) (Table 1, Supplemental Table S1). Among patients with SLE, 236 patients (84.3%) were female, with a mean (SD) age at diagnosis of 35.6 (16.0) years. The majority of clinically diagnosed SLE patients met established classification criteria: 244 (87.1%) fulfilled the EULAR/ACR classification criteria, and 240 (85.7%) were classified as SLE by the SLERPI, with mean (SD) scores of 16.4 (6.7) and 11.0 (4.0), respectively. Arthritis - as defined by the SLERPI - was the most prevalent clinical manifestation identified in 149 patients (53%), followed by subacute cutaneous lupus erythematosus/discoid lupus erythematosus in 93 patients (33%). Lupus nephritis class III/IV was reported in 25 (8.9%) SLE patients. ANA positivity was present in 271 of 280 SLE patients (97%). Hypocomplementemia, defined as reduced C3 and/or C4 levels, was observed in 139 patients (50%). Immunological disorders, as captured by the corresponding SLERPI item, were present in 219 (78%) SLE patients.

**Table 1.** Baseline demographic characteristics and SLERPI constituent items in SLE patients and non-SLE controls. Variables are presented after application of the attribution rule. Categorical variables are presented as number and percentage; continuous variables as mean and standard deviation.

	Overall patients	Non-SLE controls	SLE patients	*p*-value
	(*n* = 436)	(*n* = 156)	(*n* = 280)	
Age, years	38.54 (15.45)	43.85 (13.52)	35.58 (15.69)	<0.001
Sex, female	330 (76%)	94 (60%)	236 (84%)	<0.001
SLERPI, units	8.09 (5.15)	2.94 (1.91)	10.96 (4.03)	<0.001
Items				
Malar rash	88 (20%)	0 (0%)	88 (31%)	<0.001
Subacute cutaneous or discoid lupus	93 (21%)	0 (0%)	93 (33%)	<0.001
Alopecia	17 (3.9%)	1 (0.6%)	16 (5.7%)	0.009
Ulcers	16 (3.7%)	1 (0.6%)	15 (5.4%)	0.012
Arthritis	269 (62%)	120 (77%)	149 (53%)	<0.001
Serositis	47 (11%)	1 (0.6%)	46 (16%)	<0.001
Leucopenia	78 (18%)	8 (5.1%)	70 (25%)	<0.001
Thrombocytopenia/autoimmune hemolytic anemia	51 (12%)	1 (0.6%)	50 (18%)	<0.001
Neurological involvement	16 (3.7%)	2 (1.3%)	14 (5.0%)	0.048
Proteinuria	52 (12%)	1 (0.6%)	51 (18%)	<0.001
ANA positivity	332 (76%)	61 (39%)	271 (97%)	<0.001
Low C3 and C4	139 (32%)	0 (0%)	139 (50%)	<0.001
Immunological disorder (anybetween antiDNA, anti-Sm or antiphospholipid antibodies)	222 (51%)	3 (1.9%)	219 (78%)	<0.001
Interstitial lung disease	5 (1.1%)	0 (0%)	5 (1.8%)	0.2

The non-SLE control group (*n* = 156) was predominantly composed of patients with ankylosing spondylitis (*n* = 50, 32%), rheumatoid arthritis (*n* = 49, 31%), and psoriatic arthritis (*n* = 40, 26%) (Supplemental Table S1). Non-SLE controls had a mean (SD) age of 43.98 (13.5) years and were 60% female (Table 1). ANA positivity was present in 61 of 156 non-SLE controls (39%).

### Performance of SLERPI for early SLE diagnosis

**Figure 1.**
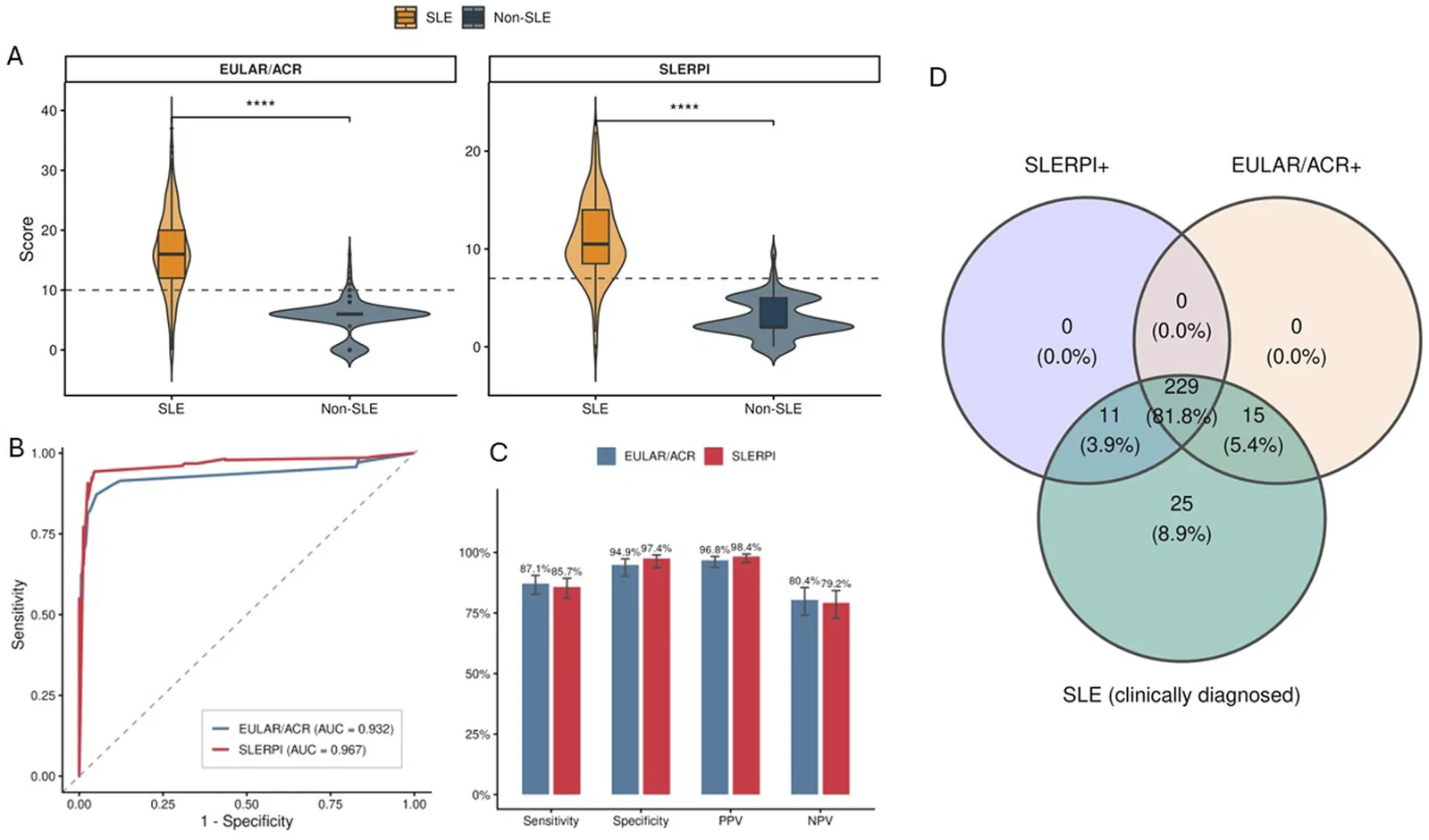
Performance of SLERPI for early SLE diagnosis. (a) Distribution of the EULAR/ACR criteria and SLERPI scores in SLE and non-SLE individuals. (b) ROC curves for SLERPI and EULAR/ACR scores in discriminating clinically diagnosed SLE from non-SLE cases. (c) Bar chart comparing sensitivity, specificity, PPV, and NPV for SLERPI and EULAR/ACR. Error bars indicate 95% confidence intervals. (d) Venn diagram illustrating the overlap between patients fulfilling SLERPI and EULAR/ACR classification criteria within a cohort of clinically diagnosed SLE. ROC: receiver operating characteristic; PPV: positive predictive value; NPV: negative predictive value; SLE: systemic lupus erythematosus.

The diagnostic performance of the SLERPI and the EULAR/ACR classification criteria was evaluated against physician diagnosis, used as the reference standard, across the full study cohort (Table 1, Table 2). In the overall cohort, the mean (SD) SLERPI score was 8.09 (5.15), being significantly higher in SLE patients compared with non-SLE controls (10.96 [SD 4.03] vs 2.94 [SD 1.91]; *p* < 0.001). Similarly, the mean (SD) EULAR/ACR score was 12.49 (7.67) overall, with SLE patients scoring markedly higher than non-SLE controls (16.40 [SD 6.66] vs 5.48 [SD 2.89]; *p* < 0.001) (Figure 1(a)).

**Table 2.** Distribution of EULAR/ACR classification criteria items in the overall population, SLE patients, and non-SLE controls. Antiphospholipid laboratory criteria include anticardiolipin antibodies, anti-β2 GP1 antibodies and lupus anticoagulant. Variables are presented after application of the attribution rule. Categorical variables are presented as number and percentage; continuous variables as mean and standard deviation.

​	Overall patients	Non-SLE controls	SLE patients	*p*-value
	(*n* = 436)	(*n* = 156)	(*n* = 280)	
EULAR/ACR, units	12.49 (7.67)	5.48 (2.89)	16.40 (6.66)	<0.001
Items				
ANA positivity	332 (76%)	61 (39%)	271 (97%)	<0.001
Fever	19 (4.4%)	5 (3.2%)	14 (5.0%)	0.4
Leucopenia	78 (18%)	8 (5.1%)	70 (25%)	<0.001
Thrombocytopenia	39 (8.9%)	1 (0.6%)	38 (14%)	<0.001
Autoimmune hemolytic anemia	24 (5.5%)	0 (0%)	24 (8.6%)	<0.001
Delirium	2 (0.5%)	0 (0%)	2 (0.7%)	0.5
Psychosis	2 (0.5%)	1 (0.6%)	1 (0.4%)	>0.9
Seizure	6 (1.4%)	0 (0%)	6 (2.1%)	0.093
Alopecia	17 (3.9%)	1 (0.6%)	16 (5.7%)	0.009
Oral ulcers	15 (3.4%)	0 (0%)	15 (5.4%)	0.003
Subacute cutaneous or discoid lupus	93 (21%)	0 (0%)	93 (33%)	<0.001
Acute cutaneous lupus	88 (20%)	0 (0%)	88 (31%)	<0.001
Pleural or pericardial effusion	47 (11%)	1 (0.6%)	46 (16%)	<0.001
Acute pericarditis	23 (5.3%)	1 (0.6%)	22 (7.9%)	0.001
Joint involvement	273 (63%)	128 (82%)	145 (52%)	<0.001
Proteinuria	52 (12%)	1 (0.6%)	51 (18%)	<0.001
Renal biopsy class II or V lupus nephritis	14 (3.2%)	0 (0%)	14 (5.0%)	0.005
Renal biopsy class III or IV lupus nephritis	25 (5.7%)	0 (0%)	25 (8.9%)	<0.001
Positive anti-cardiolipin antibodies	43 (11%)	0 (0%)	43 (15%)	<0.001
Positive anti-β2 GP1 antibodies	29 (7.5%)	0 (0%)	29 (10%)	<0.001
Positive lupus anticoagulant	48 (12%)	0 (0%)	48 (17%)	<0.001
Positive for ≥1 antiphospholipid laboratory criterion	76 (17%)	0 (0%)	76 (27%)	<0.001

The SLERPI achieved an AUC of 0.967 (95% CI 0.950-0.984), and the EULAR/ACR criteria achieved an AUC of 0.932 (95% CI 0.908-0.957). The difference between the two AUCs was statistically significant (DeLong *p* = 0.0004) (Figure 1(b)).

At a threshold score of >7, the SLERPI demonstrated a sensitivity of 85.7% (95% CI 81.1-89.3%), a specificity of 97.4% (95% CI 93.6-99.0%), a PPV of 98.4% (95% CI 95.9-99.4%), and an NPV of 79.2% (95% CI 72.9-84.3%). The EULAR/ACR classification criteria (score ≥10) yielded a sensitivity of 87.1% (95% CI 82.7-90.6%), a specificity of 94.9% (95% CI 90.2-97.4%), a PPV of 96.8% (95% CI 93.9-98.4%), and an NPV of 80.4% (95% CI 74.1-85.5%) (Figure 1(c)).

Given the observed demographic imbalance between SLE patients and controls, a sensitivity analysis was performed to assess whether age and sex confounded the diagnostic performance estimates. After adjustment for age and sex, the SLERPI’s AUC was essentially unchanged (unadjusted 0.967, 95% CI 0.950-0.984; age/sex-adjusted 0.969, 95% CI 0.954-0.985; DeLong *p* = 0.32), and the EULAR/ACR criteria’s AUC increased slightly (unadjusted 0.932, 95% CI 0.908–0.957; age/sex-adjusted 0.949, 95% CI 0.928-0.970; DeLong *p* = 0.008). In both adjusted models, the classification score remained a strong, independent predictor of SLE classification (SLERPI: adjusted OR 2.41 per unit, 95% CI 2.04-2.94, *p* < 0.001; EULAR/ACR: adjusted OR 1.60 per unit, 95% CI 1.47-1.78, *p* < 0.001). Age was independently associated with SLE diagnosis in both models, whereas sex was independently associated with diagnosis only in the EULAR/ACR model (Supplemental Tables S2 and S3).

### Discordant classification between SLERPI and EULAR/ACR classification criteria

Among the 280 SLE patients, discordant classification was observed in 26 patients (9.3%): 15 (5.4%) were EULAR/ACR-positive but SLERPI-negative (EULAR-ACR^+^/SLERPI^-^), and 11 (3.9%) were SLERPI-positive but EULAR/ACR-negative (EULAR-ACR^-^/SLERPI^+^) (Figure 1(d), Table 3, Supplemental Table S4).

**Table 3.** Baseline demographic and SLERPI constituent items according to patient category. Categorical variables are presented as number and percentage; continuous variables as mean and standard deviation.

​	EULAR-ACR+/SLERPI-	EULAR-ACR-/SLERPI-	EULAR-ACR+/SLERPI+	EULAR-ACR-/SLERPI+	*p*-value
	(*n* = 15)	(*n* = 25)	(*n* = 229)	(*n* = 11)	
Age, years	37.33 (13.54)	36.16 (19.78)	35.51 (15.44)	33.36 (15.09)	0.8
Sex, female	11 (73%)	21 (84%)	193 (84%)	11 (100%)	0.4
SLERPI, units	6.67 (0.65)	4.24 (2.23)	12.09 (3.41)	8.59 (0.74)	<0.001
Items					
Malar rash	0 (0%)	1 (4.0%)	83 (36%)	4 (36%)	<0.001
Discoid lupus	1 (6.7%)	3 (12%)	85 (37%)	4 (36%)	0.006
Alopecia	0 (0%)	0 (0%)	15 (6.6%)	1 (9.1%)	0.4
Ulcers	0 (0%)	1 (4.0%)	14 (6.1%)	0 (0%)	>0.9
Arthritis	6 (40%)	7 (28%)	136 (59%)	0 (0%)	<0.001
Serositis	5 (33%)	1 (4.0%)	40 (17%)	0 (0%)	0.037
Leucopenia	4 (27%)	4 (16%)	57 (25%)	5 (45%)	0.3
Thrombocytopenia/autoimmune hemolytic anemia	0 (0%)	0 (0%)	48 (21%)	2 (18%)	0.005
Neurological involvement	0 (0%)	0 (0%)	12 (5.2%)	2 (18%)	0.2
Proteinuria	0 (0%)	0 (0%)	50 (22%)	1 (9.1%)	0.003
ANA positivity	15 (100%)	17 (68%)	228 (100%)	11 (100%)	<0.001
Low C3 and C4	3 (20%)	1 (4.0%)	132 (58%)	3 (27%)	<0.001
Immunological disorder (any between anti-DNA, anti-Sm, antiphospholipid antibodies)	9 (60%)	9 (36%)	197 (86%)	4 (36%)	<0.001
Interstitial lung disease	1 (6.7%)	1 (4.0%)	3 (1.3%)	0 (0%)	0.2

The EULAR-ACR^+^/SLERPI^-^ group showed a higher prevalence of serositis (33% vs 17% in concordant positives, *p* = 0.037). In contrast, arthritis was less frequent in this group (40% vs 59%, *p* < 0.001), and proteinuria or thrombocytopenia/autoimmune hemolytic anemia were absent. Hypocomplementemia was present in 20.0% and immunological abnormalities in 60.0% were lower than in the concordantly positive group.

In contrast, the EULAR-ACR^-^/SLERPI^+^ group presented with a distinct phenotype characterized by the absence of both serositis and arthritis, and a higher frequency of leucopenia (45.5% vs 24.9% in concordant positives). Cutaneous features were prominent, with malar rash and subacute cutaneous or discoid lupus erythematosus each present in 36.4% of patients. Hypocomplementemia (27.3%) and immunological abnormalities (36.4%) were less frequent than in the concordantly positive group.

A subset of 25 patients (8.9%) with clinically diagnosed SLE did not meet classification thresholds for either the SLERPI or the EULAR/ACR classification criteria (EULAR-ACR^-^/SLERPI^-^). This group was predominantly female (84.0%) and characterized by a low rate of ANA positivity (68.0%). Clinical manifestations were sparse across all domains: arthritis was present in 28.0%, subacute cutaneous or discoid lupus erythematosus in 12.0%, and malar rash in 4.0%, with serositis recorded in a single patient only (4.0%). Immunological abnormalities were similarly infrequent, with hypocomplementemia in only 4.0% and anti-dsDNA, anti-Sm, or antiphospholipid antibodies in 36.0% of patients. Proteinuria, thrombocytopenia, autoimmune hemolytic anemia, and neuropsychiatric involvement were entirely absent.

Among the 156 non-SLE controls, 150 (96.2%) were correctly classified as non-SLE by both instruments. No control was misclassified as SLE by SLERPI alone; two controls (1.3%) were misclassified as SLE by the EULAR/ACR criteria only, and four controls (2.6%) were misclassified by both instruments (Supplemental Table S5 and S6).

Collectively, the SLERPI demonstrated an advantage in identifying cutaneous-predominant and leucopenia-associated presentations, whereas the EULAR/ACR criteria appeared more sensitive to serositis-driven disease. Patients negative by both instruments represented a clinically and serologically sparse subset consistent with early or undifferentiated disease.

### Potential of SLERPI for flare risk stratification

**Figure 2.**
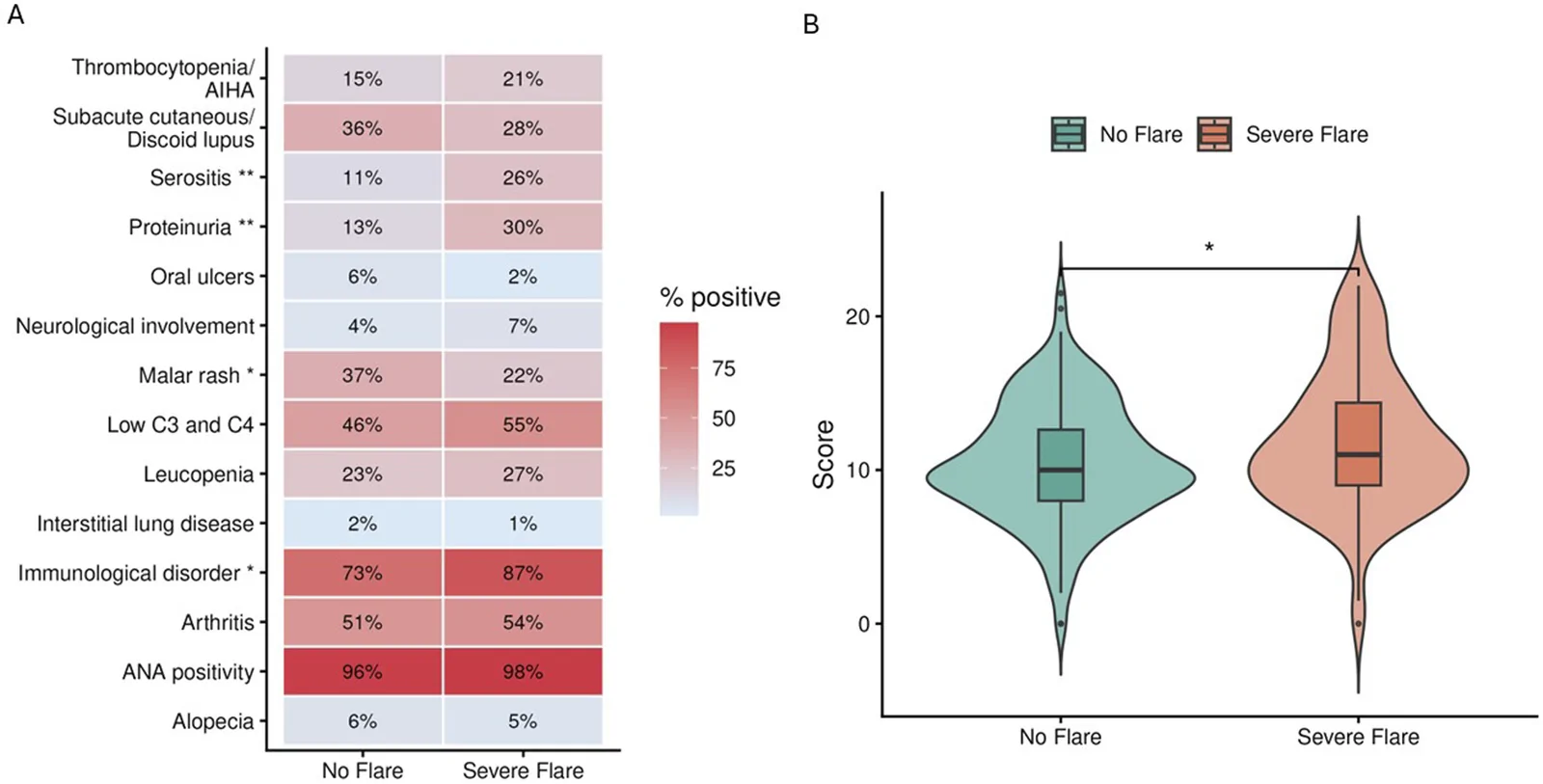
SLERPI score and severe flare. (a) Heatmap showing the prevalence of SLERPI constituent items in SLE patients according to severe flare status within 3 years of diagnosis. Colour intensity reflects the proportion of positive cases, ranging from light blue (low prevalence) to dark red (high prevalence), as indicated by the scale bar. Items are defined according to the original SLERPI scoring framework across its 14 constituent domains. Statistical significance of between-group differences was assessed by chi-square test and is indicated directly on the item label: *p < 0.05; **p < 0.01; ***p < 0.001. (b) Distribution of SLERPI scores according to severe flare status. AIHA: autoimmune hemolytic anemia; ANA: antinuclear antibody.

To explore the prognostic utility of the SLERPI beyond diagnosis, we examined whether baseline SLERPI features were associated with the occurrence of a severe flare within 3 years of diagnosis, among the 258 SLE patients with available follow-up data (Figure 2(a)). Patients excluded from the prognostic analyses due to missing flare follow-up data (*n* = 22) did not differ significantly from those included (n = 258) across baseline demographic, clinical, and serological variables (Supplemental Table S7).

Patients who developed a severe flare had significantly higher baseline SLERPI score compared with those who remained severe flare-free (11.77 [SD 4.47] vs 10.47 [SD 3.84], *p* = 0.047) (Figure 2(b)). When the continuous SLERPI total score was used as a single predictor, the Youden’s J method identified a potentially informative prognostic threshold of ≥10.2 points (Supplemental Figure 1), at which AUC was 0.576 (95% CI 0.501-0.652).

**Figure 3.**
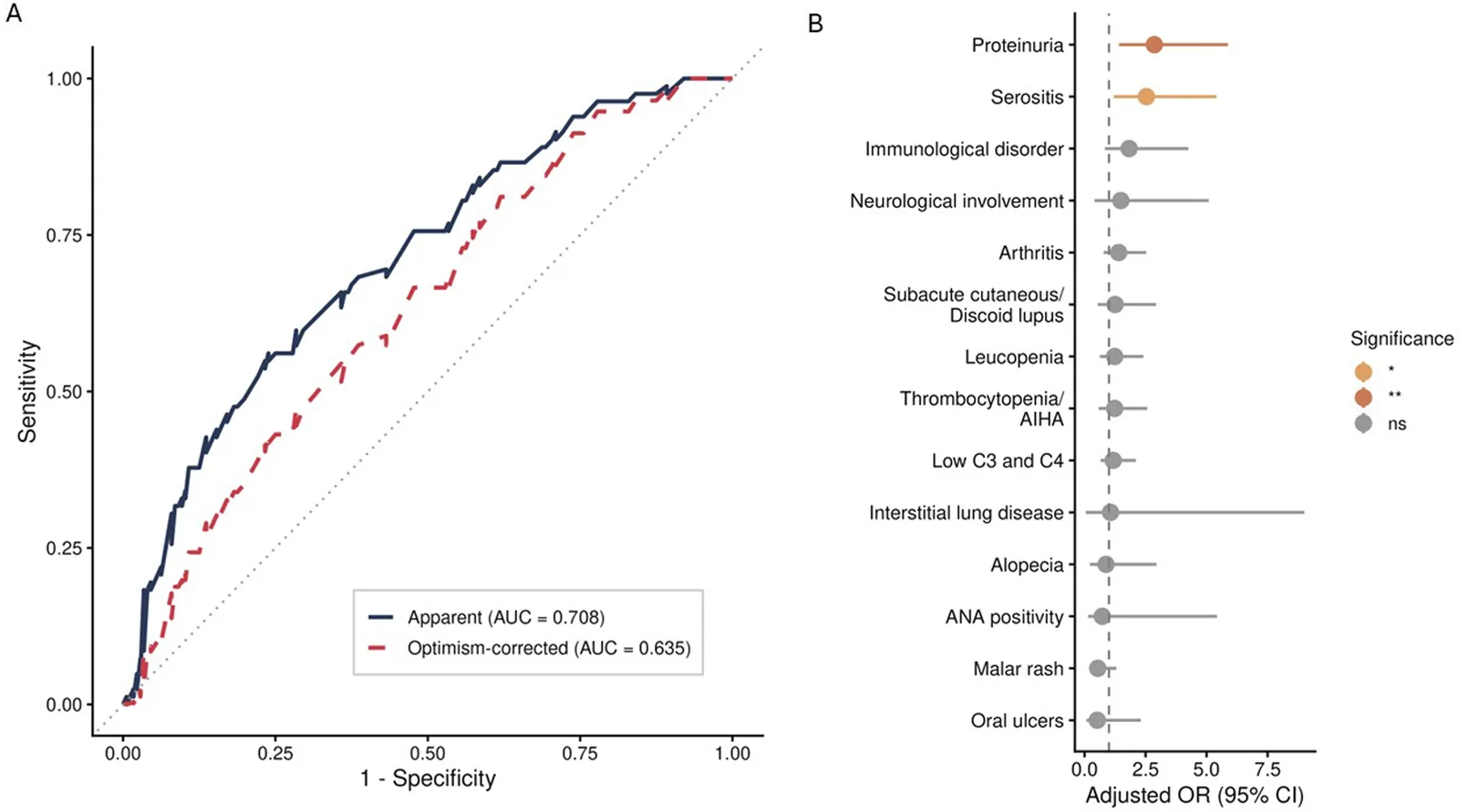
Discriminative performance of the multivariate SLERPI item model for predicting severe flare. (a) ROC curves for the multivariable logistic regression model incorporating all SLERPI items with non-zero variance as predictors of severe flare within 3 years of diagnosis. The solid navy curve represents the model’s apparent discrimination (AUC 0.708); the dashed red curve represents the optimism-corrected discrimination (AUC 0.635) obtained via bootstrap internal validation. (b) Forest plot showing adjusted OR with 95% CI derived from multivariate logistic regression including all SLERPI items with non-zero variance. The vertical dashed line represents the OR = 1. Colour denotes statistical significance after multivariable adjustment: dark red (**), *p* < 0.01; orange (*), *p* < 0.05; grey (ns), not statistically significant. ROC: receiver operating characteristic; AUC: area under the curve; OR: odds ratios; CI: confidence intervals.

A multivariate logistic regression model incorporating all SLERPI items with non-zero variance achieved an AUC of 0.708 (95% CI 0.641-0.775%) for predicting severe flare occurrence (Figure 3(a)). The events-per-predictor ratio was 5.9. Following bootstrap internal validation, the optimism-corrected AUC was 0.635 (95% CI 0.574-0.695) (Figure 3(a), Supplemental Figure 2). At the level of individual SLERPI items, proteinuria (adjusted OR 2.86, 95% CI 1.41-5.87; *p* = 0.004) and serositis (adjusted OR 2.53, 95% CI 1.20-5.41; *p* = 0.015) were independently associated with severe flare occurrence (Figure 3(b)).

To determine which components of the SLERPI item model were driving its discriminative performance, we compared the full 14-item model against a series of reduced models built from subsets of the same candidate predictors. A parsimonious model including only proteinuria and serositis - the two variables retaining independent significance in the full model-demonstrated similar performance, with an apparent AUC of 0.639 (95% CI 0.576-0.703) and an optimism-corrected AUC of 0.633. Comparable discrimination was observed for the EULAR/ACR score when modeled as a single continuous predictor (apparent AUC 0.608, 95% CI 0.532-0.683; optimism-corrected AUC 0.607).

## Discussion

In this retrospective cohort study of 436 patients evaluated at a single European tertiary center, we demonstrated that the SLERPI performs with high diagnostic accuracy in distinguishing SLE from other rheumatic diseases, with a higher AUC and specificity than the EULAR/ACR classification criteria. Beyond its diagnostic utility, we evaluated whether baseline SLERPI scores might serve as a predictor of disease trajectory.

Our findings align with and extend prior validation studies. The original SLERPI development cohort reported high accuracy across multiple patient populations, with AUC values exceeding 0.90 in the validation set.^5^ Subsequent external validations in ethnically diverse populations have reaffirmed its robust discriminatory capacity.^11,12^ Our data add to this body of evidence by demonstrating that the SLERPI retains its high performance in a retrospective Central European cohort.

The analysis of discordant classifications between the SLERPI and the EULAR/ACR criteria provides clinically meaningful insights into the phenotypic coverage of each instrument. Patients classified as SLE by the EULAR/ACR criteria but not by the SLERPI were characterized by a higher prevalence of serositis, a feature that carries significant weight in the EULAR/ACR scoring system but is not prominently represented in the SLERPI architecture. These discordance findings can be contextualized against a recent large multicenter study,^11^ which performed a detailed phenotypic analysis of discordant cases among 655 SLE patients evaluated by SLERPI, ACR-1997, Systemic Lupus International Collaborating Clinics (SLICC)-2012, and EULAR/ACR-2019 criteria, reporting that EULAR/ACR-2019 most frequently failed to classify patients with mucocutaneous-predominant presentations.

Conversely, patients classified by the SLERPI but not by the EULAR/ACR criteria exhibited a cutaneous-predominant phenotype with leucopenia, suggesting that the SLERPI captures a clinically relevant subset of patients with milder disease presentations-patients who might otherwise be missed by the EULAR/ACR criteria due to its domain-based scoring structure in which only the highest criterion in each organ domain is scored. These findings underscore the complementary strengths of both instruments, with their combined use enhancing diagnostic sensitivity across the SLE spectrum, particularly in early or phenotypically atypical disease.

The subset of patients who tested negative by both instruments - characterized by sparse clinical features, low ANA positivity and absence of major organ involvement - likely represents either early, mild or less differentiated disease. This population would benefit from longitudinal follow-up and repeat evaluation to ensure timely diagnosis and intervention.

A key aspect of this study is the transition from classification to individualized risk assessment, which is central to the concept of precision medicine in rheumatology.^13^ Although the SLERPI score at a ≥10.2-point threshold showed weak discriminatory capacity for predicting severe flare risk, patients who experienced a severe flare within the first 3 years of diagnosis showed a tendency toward higher baseline scores. These findings should be interpreted as hypothesis-generating, warranting further investigation of the risk-stratification potential of SLERPI.

The multivariable analysis incorporating all SLERPI items, pinpointed proteinuria and serositis as the key independent drivers of future severe flares. This finding is biologically plausible and aligns with existing literature linking renal involvement and inflammatory serositis with a more aggressive disease course in SLE.^14,15,16^ Notwithstanding these associations, internal bootstrap validation demonstrated appreciable model optimism. Although the apparent discrimination was moderate (AUC 0.708), the optimism-corrected AUC declined to 0.635, indicating only modest predictive performance after adjustment for overfitting. Comparison with reduced models suggested that this discriminative ability was largely driven by proteinuria and serositis, and the overall predictive value of the full SLERPI-item model, as well as its added value beyond these two variables, therefore appears limited.

Several limitations of the study should be acknowledged. First, the retrospective design introduces the possibility of incomplete data capture. Second, flare ascertainment and SLERPI scoring were performed by the same study team assessor(s) without formal blinding, which introduces the potential for assessment bias. Third, missing follow-up data were handled via complete-case exclusion; although excluded and included patients did not differ significantly across the variables assessed, residual confounding cannot be entirely excluded. Finally, the SLERPI was originally developed and validated primarily as a diagnostic tool; its repurposing for prognostic stratification, while conceptually sound, requires prospective validation.

In conclusion, this study confirms that the SLERPI performs with strong diagnostic accuracy in a single-centre retrospective Central European SLE cohort, achieving a higher AUC and specificity than the EULAR/ACR classification criteria.

## Supplemental material

Suppplemental material - Validation and prognostic utility of SLERPI in a single-centre retrospective Central European cohort Suppplemental material for Validation and prognostic utility of SLERPI in a single-centre retrospective Central European cohort by Andrea Zoli, Seda Nur Aydogdu, Danae-Mona Nöthling, Paraskevi Chasani, Maria Antonietta D' Agostino, Silvia Laura Bosello, Andreas Ramming, Christina Bergmann, Georg Schett, Chiu Wai Shirley Chan, Panagiotis Garantziotis in Lupus.

## Data Availability

All data generated or analyzed during this study are included in this published article [and its supplemental information files].*
