# Supplementary material for: Validation and prognostic utility of SLERPI in a single-centre retrospective Central European cohort
Source: Lupus. 2026 Aug 5;35(12):1292–300. doi: 10.1177/09612033261474985 (PMC13569890; doi:10.1177/09612033261474985)
Supplement: Supplementary file 1 [file sj-pdf-1-lup-10.1177_09612033261474985.pdf]

Supplemental appendix for:

Validation and prognostic utility of SLERPI in a real-world European cohort

Supplemental Table S1. Diagnostic composition of the non-SLE control group.

Diagnosis	n (%)
Ankylosing spondylitis	50 (32%)
Rheumatoid arthritis	49 (31%)
Psoriatic arthritis	40 (26%)
Adult-onset Still's disease	8 (5.1%)
Systemic sclerosis	5 (3.2%)
Inflammatory bowel disease-related arthritis	2 (1.3%)
Behçet's disease	1 (0.6%)
Gout	1 (0.6%)

Supplemental Table S2. Sensitivity analysis assessing the effect of age and sex on the diagnostic performance of the SLERPI. OR, Odds ratios (OR); CI, confidence intervals.

	OR	95% CI lower	95% CI upper	p-value
SLERPI score (per unit)	2.41	2.042	2.943	<0.001
Age (per year)	0.967	0.941	0.992	0.011
Sex (male)	0.633	0.269	1.471	0.288

Supplemental Table S3. Sensitivity analysis assessing the effect of age and sex on the diagnostic performance of the EULAR/ACR classification criteria. OR, Odds ratios (OR); CI, confidence intervals.

	OR	95% CI lower	95% CI upper	p-value
EULAR/ACR score (per unit)	1.601	1.465	1.776	<0.001
Age (per year)	0.955	0.933	0.976	<0.001
Sex (male)	0.334	0.154	0.694	0.004

Supplemental Table S4. EULAR/ACR classification criteria items according to patient category. Antiphospholipid laboratory criteria include anticardiolipin antibodies, anti-β2 GP1 antibodies and lupus anticoagulant. Categorical variables are presented as number and percentage; continuous variables as mean and standard deviation.

	**EULAR-ACR+/SLERPI-**	**EULAR-ACR-/ SLERPI-**	**EULAR-ACR+/ SLERPI+**	**EULAR-ACR-/ SLERPI+**	**p-value**
	(n = 15)	(n = 25)	(n = 229)	(n = 11)	
**EULAR/ACR (units)**	12.80 (2.27)	4.80 (3.27)	18.35 (5.38)	7.00 (2.05)	<0.001

Items					
**ANA positivity**	15 (100%)	17 (68%)	228 (100%)	11 (100%)	<0.001
**Fever**	1 (6.7%)	1 (4.0%)	12 (5.2%)	0 (0%)	>0.9
**Leucopenia**	4 (27%)	4 (16%)	57 (25%)	5 (45%)	0.3
**Thrombocytopenia**	1 (6.7%)	0 (0%)	35 (15%)	2 (18%)	0.10
**Autoimmune hemolytic anemia**	0 (0%)	0 (0%)	23 (10%)	1 (9.1%)	0.2
**Delirium**	0 (0%)	0 (0%)	2 (0.9%)	0 (0%)	> 0.9
**Psychosis**	0 (0%)	0 (0%)	1 (0.4%)	0 (0%)	>0.9
**Seizure**	0 (0%)	0 (0%)	5 (2.2%)	1 (9.1%)	0.4
**Alopecia**	0 (0%)	0 (0%)	15 (6.6%)	1 (9.1%)	0.4
**Mucosal ulcers**	0 (0%)	1 (4.0%)	14 (6.1%)	0 (0%)	>0.9
**Subacute cutaneous or discoid lupus**	1 (6.7%)	3 (12%)	85 (37%)	4 (36%)	0.006
**Acute cutaneous lupus**	0 (0%)	1 (4.0%)	83 (36%)	4 (36%)	<0.001
**Pleural or pericardial effusion**	5 (33%)	1 (4.0%)	40 (17%)	0 (0%)	0.037
**Acute pericarditis**	3 (20%)	1 (4.0%)	18 (7.9%)	0 (0%)	0.2
**Joint involvement**	6 (40%)	5 (20%)	134 (59%)	0 (0%)	<0.001
**Proteinuria > 0.5 g/24 h**	0 (0%)	0 (0%)	50 (22%)	1 (9.1%)	0.003
**Renal biopsy class II or V renal nephritis**	0 (0%)	0 (0%)	14 (6.1%)	0 (0%)	0.7
**Renal biopsy class III or IV lupus nephritis**	0 (0%)	0 (0%)	25 (11%)	0 (0%)	0.2

**Positive anti-cardiolipin antibodies**	3 (20%)	1 (4.0%)	38 (17%)	1 (9.1%)	0.3
**Positive anti-β2 GP1 antibodies**	2 (13%)	1 (4.0%)	26 (11%)	0 (0%)	0.5
**Positive lupus anticoagulant**	4 (27%)	3 (12%)	38 (17%)	3 (27%)	0.5
**Positive for ≥1 antiphospholipid laboratory criterion**	5 (33%)	4 (16%)	64 (28%)	3 (27%)	0.6

Supplemental Table S5. Classification of non-SLE controls by SLERPI criteria. Categorical variables are presented as number (%); continuous variables as mean (standard deviation). ANA, antinuclear antibody.

	**EULAR-ACR-/ SLERPI-**	**EULAR-ACR+/ SLERPI-**	**EULAR-ACR+/ SLERPI+**
	(n = 150)	(n = 2)	(n = 4)
**Age, years**	44.01 (13.52)	30.00 (19.80)	44.75 (10.53)
**Sex, female**	88 (59%)	2 (100%)	4 (100%)
**SLERPI, units**	2.77 (1.69)	4.75 (0.35)	8.50 (1.15)
**Items**			
**Malar rash**	0 (0%)	0 (0%)	0 (0%)
**Subacute cutaneous or discoid lupus**	0 (0%)	0 (0%)	0 (0%)
**Alopecia**	1 (0.7%)	0 (0%)	0 (0%)
**Ulcers**	1 (0.7%)	0 (0%)	0 (0%)

**Arthritis**	115 (77%)	1 (50%)	4 (100%)
**Serositis**	1 (0.7%)	0 (0%)	0 (0%)
**Leucopenia**	7 (4.7%)	1 (50%)	0 (0%)
**Thrombocytopenia / autoimmune hemolytic anemia**	0 (0%)	0 (0%)	1 (25%)
**Neurological involvement**	2 (1.3%)	0 (0%)	0 (0%)
**Proteinuria**	0 (0%)	0 (0%)	1 (25%)
**ANA positivity**	55 (37%)	2 (100%)	4 (100%)
**Low C3 and C4**	0 (0%)	0 (0%)	0 (0%)
**Immunological disorder (any between anti-DNA, anti-Sm, antiphospholipid antibodies)**	1 (0.7%)	0 (0%)	2 (50%)
**Interstitial lung disease**	0 (0%)	0 (0%)	0 (0%)

Supplemental Table S6. Classification of non-SLE controls by EULAR/ACR classification criteria. Antiphospholipid laboratory criteria include anticardiolipin antibodies, anti-β2 GP1 antibodies and lupus anticoagulant. Categorical variables are presented as number (%); continuous variables as mean (standard deviation). ANA, antinuclear antibody.

	**EULAR-ACR-/**	**EULAR-ACR+/SLERPI-**	**EULAR-ACR+/ SLERPI+**
	(n = 150)	(n = 2)	(n = 4)

**EULAR/ACR, units**	5.24	10.50 (0.71)	12.00 (2.83)
**Items**			
**ANA positivity**	55 (37%)	2 (100%)	4 (100%)
**Fever**	4 (2.7%)	1 (50%)	0 (0%)
**Leucopenia**	7 (4.7%)	1 (50%)	0 (0%)
**Thrombocytopenia**	0 (0%)	0 (0%)	1 (25%)
**Autoimmune hemolytic anemia**	0 (0%)	0 (0%)	0 (0%)
**Delirium**	0 (0%)	0 (0%)	0 (0%)
**Psychosis**	1 (0.7%)	0 (0%)	0 (0%)
**Seizure**	0 (0%)	0 (0%)	0 (0%)
**Alopecia**	1 (0.7%)	0 (0%)	0 (0%)
**Oral ulcers**	0 (0%)	0 (0%)	0 (0%)
**Subacute cutaneous or discoid lupus**	0 (0%)	0 (0%)	0 (0%)
**Acute cutaneous lupus**	0 (0%)	0 (0%)	0 (0%)
**Pleural or pericardial effusion**	1 (0.7%)	0 (0%)	0 (0%)
**Acute pericarditis**	1 (0.7%)	0 (0%)	0 (0%)
**Joint involvement**	122	2 (100%)	4 (100%)
**Proteinuria**	0 (0%)	0 (0%)	1 (25%)
**Renal biopsy class II or V lupus nephritis**	0 (0%)	0 (0%)	0 (0%)
**Renal biopsy class III or IV lupus nephritis**	0 (0%)	0 (0%)	0 (0%)
**Positive anti-cardiolipin antibodies**	0 (0%)	0 (0%)	0 (0%)
**Positive anti-β2 GP1 antibodies**	0 (0%)	0 (0%)	0 (0%)
**Positive lupus anticoagulant**	0 (0%)	0 (0%)	0 (0%)
**Positive for ≥1 antiphospholipid laboratory criterion**	0 (0%)	0 (0%)	0 (0%)

Supplemental Table S7. Baseline characteristics of SLE patients included in versus excluded from the flare prognostic analysis. Antiphospholipid laboratory criteria include anticardiolipin antibodies, anti-β2 GP1 antibodies and lupus anticoagulant. Categorical variables are presented as number (%); continuous variables as mean (standard deviation). Between-group

comparisons were performed using the chi-square test (categorical variables) or Mann-Whitney U test (continuous variables). ANA, antinuclear antibody.

	**Overall**	**Excluded**	**Included**	**p-value**
	(n = 280)	(n = 22)	(n = 258)	
**Age, years**	36 (16)	39 (16)	35 (16)	0.4
**Sex, female**	236 (84%)	20 (91%)	216 (84%)	0.5
**SLERPI, units**	11.0 (4.0)	11.9 (3.6)	10.9 (4.1)	0.3
**EULAR/ACR, units**	16 (7)	18 (7)	16 (7)	0.3
**SLERPI items**				
**Malar rash**	88 (31%)	5 (23%)	83 (32%)	0.4
**Subacute cutaneous / discoid lupus**	93 (33%)	6 (27%)	87 (34%)	0.5
**Alopecia**	16 (5.7%)	1 (4.5%)	15 (5.8%)	>0.9
**Ulcers**	15 (5.4%)	3 (14%)	12 (4.7%)	0.10
**Arthritis**	149 (53%)	15 (68%)	134 (52%)	0.14
**Serositis**	46 (16%)	6 (27%)	40 (16%)	0.2
**Leucopenia**	70 (25%)	8 (36%)	62 (24%)	0.2
**Thrombocytopenia / autoimmune hemolytic anemia**	50 (18%)	6 (27%)	44 (17%)	0.2
**Neurological involvement**	14 (5.0%)	0 (0%)	14 (5.4%)	0.6
**Proteinuria**	51 (18%)	3 (14%)	48 (19%)	0.8
**ANA positivity**	271 (97%)	22 (100%)	249 (97%)	>0.9
**Low C3 and/or C4**	139 (50%)	13 (59%)	126 (49%)	0.4
**Immunological disorder (any between anti-dsDNA, anti-Sm, or**	219 (78%)	19 (86%)	200 (78%)	0.4

	Overall	Excluded	Included	p-value
**antiphospholipid antibodies)**				
**Interstitial lung disease**	5 (1.8%)	1 (4.5%)	4 (1.6%)	0.3
**EULAR/ACR items**				
**ANA positivity**	271 (97%)	22 (100%)	249 (97%)	>0.9
**Fever**	14 (5.0%)	1 (4.5%)	13 (5.0%)	>0.9
**Leucopenia**	70 (25%)	8 (36%)	62 (24%)	0.2
**Thrombocytopenia**	38 (14%)	3 (14%)	35 (14%)	>0.9
**Autoimmune hemolytic anemia**	24 (8.6%)	3 (14%)	21 (8.1%)	0.4
**Delirium**	2 (0.7%)	0 (0%)	2 (0.8%)	>0.9
**Psychosis**	1 (0.4%)	0 (0%)	1 (0.4%)	>0.9
**Seizure**	6 (2.1%)	0 (0%)	6 (2.3%)	>0.9
**Alopecia**	16 (5.7%)	1 (4.5%)	15 (5.8%)	>0.9
**Oral ulcers**	15 (5.4%)	3 (14%)	12 (4.7%)	0.10
**Subacute cutaneous or discoid lupus**	93 (33%)	6 (27%)	87 (34%)	0.5
**Acute cutaneous lupus**	88 (31%)	5 (23%)	83 (32%)	0.4
**Pleural or pericardial effusion**	46 (16%)	6 (27%)	40 (16%)	0.2
**Acute pericarditis**	22 (7.9%)	1 (4.5%)	21 (8.1%)	>0.9
**Joint involvement**	145 (52%)	15 (68%)	130 (50%)	0.11
**Proteinuria**	51 (18%)	3 (14%)	48 (19%)	0.8
**Renal biopsy class II or V lupus nephritis**	14 (5.0%)	1 (4.5%)	13 (5.0%)	>0.9

	Overall	Excluded	Included	p-value
**Renal biopsy class III or IV lupus nephritis**	25 (8.9%)	1 (4.5%)	24 (9.3%)	0.7
**Positive anti-cardiolipin antibodies**	43 (15%)	5 (23%)	38 (15%)	0.4
**Positive anti-β2GP1 antibodies**	29 (10%)	4 (18%)	25 (9.7%)	0.3
**Positive lupus anticoagulant**	48 (17%)	5 (23%)	43 (17%)	0.6
**Positive for ≥1 antiphospholipid laboratory criterion**	76 (27%)	7 (32%)	69 (27%)	0.6

Supplemental Figure 1. Optimal cut-off of SLERPI for predicting severe flare. Plot of Youden's J across all possible SLERPI thresholds. The dashed vertical line indicates the optimal cut-off, while the dotted line represents the predefined diagnostic threshold.

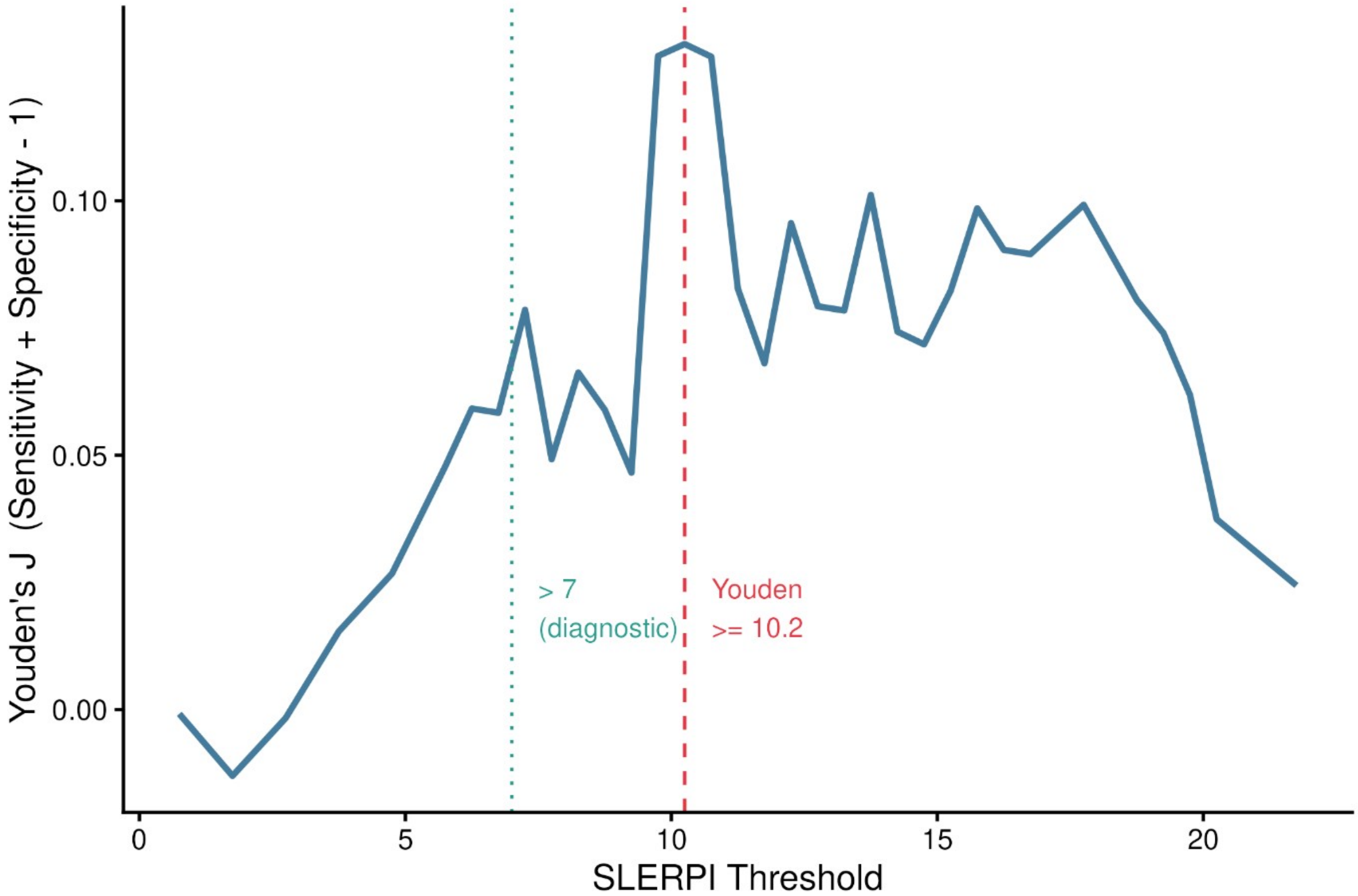

Supplemental Figure 2. Bootstrap-corrected calibration of the multivariable SLERPI-item model for severe flare prediction. Calibration plot comparing predicted and observed probabilities of severe flare within three years of diagnosis, derived from the multivariable logistic regression model incorporating all SLERPI items with non-zero variance. The dotted line represents the model's apparent calibration; the solid black line represents the bias-corrected calibration curve obtained via bootstrap resampling (1,000 replicates, Harrell's method), with grey lines indicating the 95% confidence limits around this curve. C.L., confidence limits.

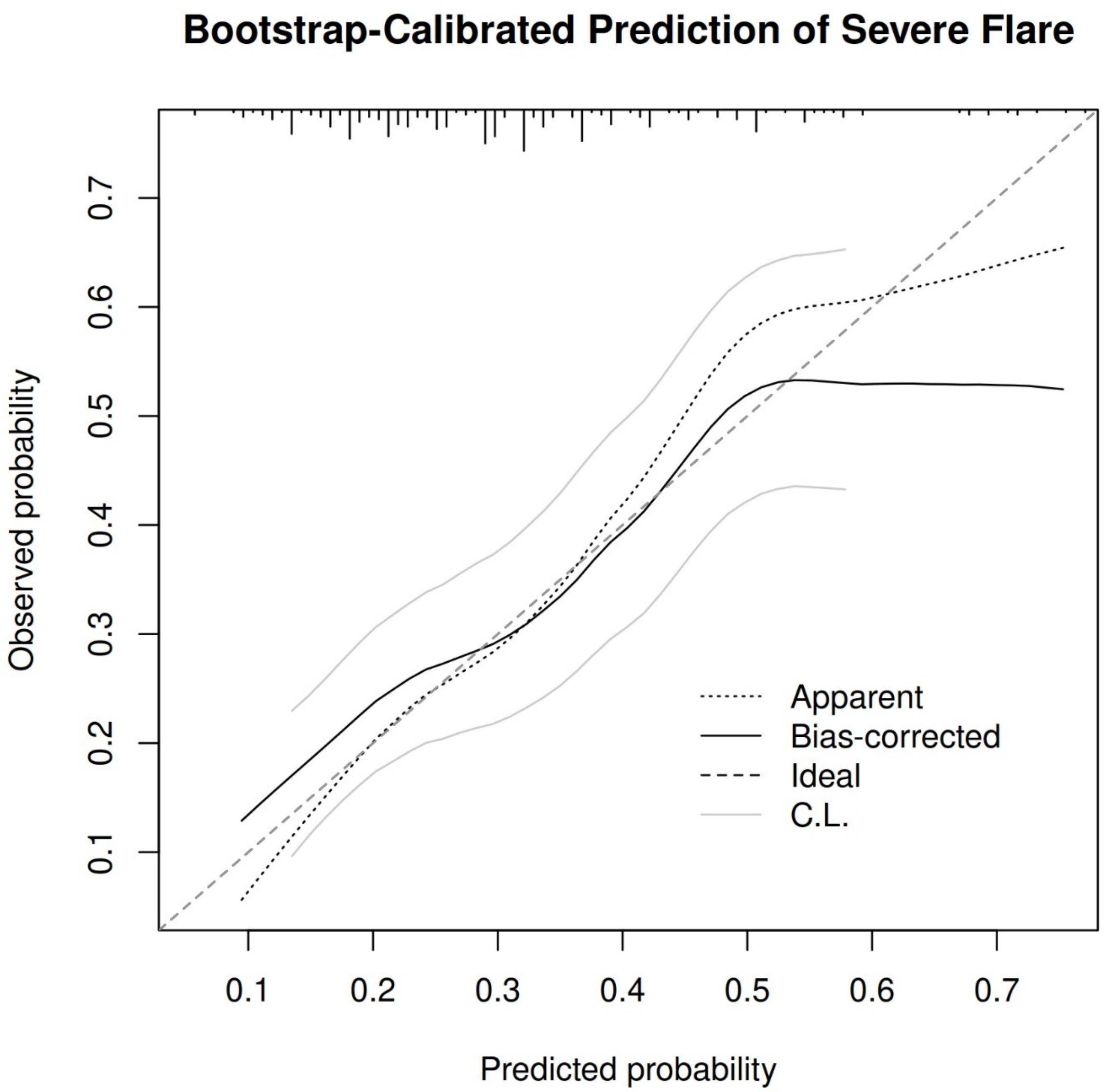
Bootstrap-Calibrated Prediction of Severe Flare
Observed probability
Predicted probability
0.1
0.2
0.3
0.4
0.5
0.6
0.7
Apparent
Bias-corrected
Ideal
C.L.
